# A Study of the Direct Effect of Pegylated Graphene Oxide Nanoparticles and Fullerenol C_60_(OH)_24_ on the Differentiation of Regulatory T Cells In Vitro

**DOI:** 10.3390/nano16110667

**Published:** 2026-05-26

**Authors:** Svetlana Zamorina, Darya Usanina, Kseniya Devyatova, Maria Bochkova, Maria Nikitina, Mikhail Rayev, Valeria Timganova

**Affiliations:** 1 Branch of the Perm Federal Research Center, Ural Branch of the Russian Academy of Sciences, Institute of Ecology and Genetics of Microorganisms, Goleva St., 13, Perm 614081, Russia; zamorina.sa@gmail.com (S.Z.); usanina_d@mail.ru (D.U.); krasnykh-m@mail.ru (M.B.); kropanevamasha@gmail.com (M.N.); mraev@iegm.ru (M.R.); 2 Department of Microbiology and Immunology, Faculty of Biology, Perm State National Research University, Bukireva St., 15, Perm 614990, Russia; xdevyatova@gmail.com

**Keywords:** regulatory T cells, PEGylated graphene oxide, fullerenol C_60_(OH)_24_, biomedicine, in vitro

## Abstract

Regulatory T cells (Tregs) play a key role in immune tolerance and are promising targets for treating immune-mediated diseases. This study investigated the direct effects of PEGylated graphene oxide nanoparticles (LP-GO, BP-GO at 5–25 μg/mL) and fullerenol C_60_(OH)_24_ (25–200 μg/mL) on human Treg viability and differentiation in vitro. Tregs were induced from peripheral blood CD4^+^ T cells using IL-2, TGF-β, and CD2/CD3/CD28 activation beads for 72 h with nanoparticles. Assessments included viability, apoptosis (Zombie aqua/Annexin V), phenotype (CD45^+^CD4^+^CD25^+^CD127^dim/−^FOXP3^+^), nanoparticle sorption (intrinsic fluorescence), and IL-10 production. Neither PEGylated graphene oxide nor fullerenol C_60_(OH)_24_ affected T-helper (CD4^+^) viability (95.35–96.15%) nor early/late apoptosis levels. Despite this, we found a decrease in the percentage of CD4^+^ cells in cultures exposed to 50–200 μg/mL of fullerenol C_60_(OH)_24_. The percentage and absolute number of Treg cells decreased with 100–200 μg/mL of fullerenol, while IL-10 levels declined following treatment with 200 μg/mL of the same nanoparticles. Graphene oxide nanoparticles showed virtually no localization within or on cells. However, T helper and Treg cells demonstrated concentration-dependent sorption of fullerenol C_60_(OH)_24_ at concentrations of 100–200 μg/mL without a reduction in viability. These findings demonstrate good in vitro biocompatibility of the nanoparticles at pharmacological concentrations up to 25 μg/mL, alongside the inhibition of Treg differentiation with 100–200 μg/mL of fullerenol C_60_(OH)_24_.

## 1. Introduction

The main functions of regulatory T cells (Tregs) involve suppressing inflammation, regulating immune responses, and maintaining a balance between different arms of the immune system. Due to these properties, Tregs play a crucial role in preventing autoreactivity, promoting the development of immune tolerance to self-antigens, and protecting the fetus from attacks by maternal immune cells during pregnancy [1]. Regulatory T lymphocytes influence a wide range of immune cells, including effector T cells (CD4^+^ and CD8^+^ T cells), and antigen-presenting cells (APCs) such as dendritic cells, natural killer (NK) cells, and B cells. Recently, the role of Tregs in regulating other cell types, such as neutrophils, γδ T cells, innate lymphoid cells (ILCs) [2], and myeloid-derived suppressor cells (MDSCs) [3], has also been studied.

The suppressive action of Tregs is realized through direct cell-to-cell contact, the secretion of immunosuppressive cytokines such as interleukin-10 (IL-10) and transforming growth factor-β (TGF-β), the production of cytotoxic molecules that induce apoptosis in target cells, and the disruption of metabolic processes, such as reducing IL-2 availability for other T cells [4]. The context of the immune response, such as the type of pathogen or affected tissue, further influences the engaged regulatory mechanisms. Tregs express CD3, CD4, CD25, and FOXP3, while lacking CD127. The surface antigens CD3 and CD4 serve to identify T helper lymphocytes, from which the Treg subpopulation originates. The CD25 molecule, localized on the Treg membrane, represents the α-chain of the IL-2 receptor (IL-2R-α) and is an important marker of activation for these cells. The canonical transcription factor FOXP3 is essential for the development, maintenance, and identification of Tregs. FOXP3 deficiency leads to defects in Treg development and function, which, in turn, cause inflammatory and autoimmune diseases in both humans and mice [5,6].

Homeostatic proliferation of T helpers is supported by the cytokine IL-7 [7]. The presence of the α-chain of the IL-7 receptor (CD127) is characteristic of most conventional T helpers. However, as Tregs develop their suppressive properties, their CD127 expression decreases, and is inversely correlating with CD25 and FOXP3 levels [8,9]. Overall, these surface antigens and the FOXP3 transcription factor are sufficient for the precise identification of the Treg subpopulation. Thus, modern methods define this subpopulation as CD4^+^CD25^high^FOXP3^+^CD127^dim/−^ [10]. Surface molecules such as CTLA-4 (cytotoxic T-lymphocyte-associated protein 4), GITR (glucocorticoid-induced TNFR-related protein), and the neuropilin-1 receptor (Nrp1) are known to serve as targets for nanoparticles of various types to manipulate Tregs [11].

Thus, CTLA-4 is a key molecule for Tregs, functioning as an immune checkpoint protein that negatively regulates the activation of the general T-cell pool [12]. The GITR receptor is constitutively expressed at high levels on Tregs, acting as a costimulatory molecule capable of either inhibiting Treg function or stimulating effector T-cell function, depending on the context [13]. Notably, Nrp1 on Tregs is critically important for their function, stability, and survival, especially in the tumor microenvironment [10].

The stability of Tregs is ensured by their ability to maintain FOXP3 expression under inflammatory-stress conditions, thereby preventing their conversion into pro-inflammatory effector cells. This stability is crucial for Tregs to effectively perform their immunosuppressive functions. Metabolic pathways, including glycolysis, β-oxidation of fatty acids, and amino acid catabolism, are known to be necessary for maintaining Treg stability [14]. Moreover, metabolic pathways determine the fate of T cells; thus, high levels of glycolysis promote the differentiation of effector T cells, while fatty acid oxidation promotes Treg differentiation. These metabolic features allow Tregs to function effectively in the inflamed-tumor microenvironment, which often experiences nutrient deficiency [15]. In addition, Tregs express much lower levels of Glut1 and exhibit lower glycolysis rates than effector T helper cells [16].

As of May 2026, a significant number of clinical trials using Tregs as a treatment method have been registered on ClinicalTrials.gov, totaling over 260 entries. Notably, polyclonal Tregs cultured ex vivo have been used in the majority of these trials [14]. Consequently, the use of nanoparticles of various types represents a promising approach to advancing this biomedical field.

Nanoparticles have already yielded considerable success in biomedicine by enabling targeted drug delivery, which increases treatment efficacy and reduces side effects by concentrating the therapy on affected cells [17]. They also improve diagnostic imaging by providing clearer and more detailed images for early detection and monitoring, while offering advanced capabilities in tissue engineering and regenerative medicine. Looking ahead, future research should focus on developing multifunctional nanoparticles with combined diagnostic and therapeutic (theranostic) properties, as well as furthering the progress of personalized medicine [17].

Carbon-based nanoparticles (CBNs), including carbon nanotubes, graphene, fullerenes, and carbon dots, offer significant advantages in biomedicine due to their favorable properties, such as high biocompatibility, a large surface area for drug loading, and unique optical and mechanical characteristics. The ease of surface functionalization enables targeted drug delivery, improved bioimaging (through fluorescent or photothermal effects), and relatively low toxicity compared with many metallic analogues [18]. In particular, our research focuses on two types of CBNs: PEGylated graphene oxide (GO) and fullerenol C_60_(OH)_24_.

Graphene is an allotrope of carbon that represents a promising material for a wide range of biomedical applications due to its unique combination of properties. Numerous studies have already addressed the use of graphene and its derivatives in tissue engineering, tumor therapy, targeted drug delivery, and related fields [19]. Cytotoxicity studies have shown that graphene itself can be toxic to cells, but the functionalization of its surface with biocompatible polymers, such as polyethylene glycol (PEG), substantially reduces these negative effects [20]. This functionalization is more conveniently carried out using graphene oxide (GO) rather than pristine graphene, since the oxygen-containing groups on its surface facilitate the modification process.

Polyhydroxylated fullerenes (fullerenols) C_60_(OH)_n_ are promising carbon allotropes due to their hydrophilicity, stability, and low toxicity. Owing to their straightforward functionalization, fullerenols can be used not only for the intracellular delivery of drugs and genetic vectors, but also as vaccine adjuvants [21,22].

Studying the effects of nanoparticles on the immune system is crucial for developing safe and effective therapies and for understanding potential health risks. Nanoparticles interact with the immune system both beneficially (e.g., by enhancing immunotherapy and vaccines) and detrimentally (e.g., by inducing inflammation or immunotoxicity).

Understanding these interactions, which are dictated by the physical properties of the nanoparticles, allows researchers to design safer nanotechnologies and to develop new treatment strategies [23]. The aim of this work was to study the direct effects of PEGylated graphene oxide (GO-PEG) nanoparticles and fullerenol C_60_(OH)_24_ on the viability and differentiation of human Tregs in vitro.

To date, numerous studies have investigated the direct effects of nanoparticles on various immune cell subpopulations. However, regulatory T cells (Tregs) have been largely overlooked in this context; when mentioned, they are typically examined only in relation to the functions of antigen-presenting cells following nanoparticle exposure. Nevertheless, in any therapeutic application of nanoparticles, it is evident that Tregs will also encounter these particles, necessitating an understanding of their response to direct exposure to such foreign materials.

## 2. Materials and Methods

**Characterization of carbon nanoparticles.** In the study, graphene oxide nanoparticles with an initial size of 100–200 nm (Ossila Ltd., Sheffield, UK) were used, coated with linear (LP-GO) and branched (BP-GO) polyethylene glycol (PEG). GO nanoparticles were modified by covalently attaching amino-terminated PEG (LP-NH_2_ and BP-NH_2_) to the surface carboxyl groups of GO, forming an amide bond. The modification procedures were carried out at the Institute of Technical Chemistry, Ural Branch of the Russian Academy of Sciences, by PhD A. I. Nechaev. Changes in the structure and composition of GO after PEGylation were confirmed using UV and Fourier-transform infrared (FTIR) spectroscopy. The degree of PEG coverage on the nanoparticles was determined by thermogravimetric analysis (TGA) on a TGA/DSC 1 Mettler-Toledo instrument (Greifensee, Switzerland). A detailed description of the functionalization of the nanoparticles and the characterization of their properties has been reported in our previous work [24], and the main characteristics are provided in S1.

Fullerenol C_60_(OH)_24_ (MSTWS60-Bio, 99.99%, MST-Nano, Saint Petersburg, Russia) was also used. Physicochemical characterization of the fullerenol was performed at the Institute of Technical Chemistry, Ural Branch of the Russian Academy of Sciences, by PhD D. M. Kiselkov. IR spectroscopy confirmed that the spectral bands correspond to polyhydroxylated fullerenes, TGA/DSC analysis showed that fullerenol decomposition occurs in the range 70–830 °C, and the electronic spectrum of the aqueous solution demonstrated the absence of unreacted starting fullerene in the studied sample. A detailed characterization of the fullerenol nanoparticles used has been described previously [25], and the main characteristics are provided in S1. In Table 1, a comparative characterization of the graphene oxide and fullerenol nanoparticles used in this work is presented.

**The quantitative endotoxin content** in graphene oxide and fullerenol C_60_(OH)_24_ solutions was assessed using the LAL test (Chromogenic Endotoxin Quant Kit, Thermo Scientific, Waltham, MA, USA). It was found that at low endotoxin standard concentrations (0.01–0.1 EU/mL), less than 0.01 EU/mL (0.006 EU/mL) of endotoxin was detected in fullerenol C_60_(OH)_24_ samples, which aligns with generally accepted recommendations [26]. In LP-GO samples (5 μg/mL), the endotoxin level was 2.3 EU/mL (0.23 ng/mL); in BP-GO samples (5 μg/mL), it was 0.6 EU/mL (0.06 ng/mL).

**Justification for the nanoparticle concentrations used**. LP-GO and BP-GO were used at final concentrations of 5 and 25 μg/mL, while fullerenol C_60_(OH)_24_ was used at final concentrations of 25, 50, 100, and 200 μg/mL. Graphene oxide nanoparticle concentrations of 5 and 25 μg/mL were selected for the study, as we had previously demonstrated effects at these concentrations on the viability, polarization, and cytokine production of Th17 cells [27]. Despite the fact that Treg cells and Th17 cells play opposite roles in the immune response, they are both T helpers [28]. The range of fullerenol C_60_(OH)_24_ particle concentrations used was significantly broader, as nanoparticles had previously been shown to exert cytotoxic effects on T cells at concentrations above 100 μg/mL [29]. This work is part of a large project on the sequential study of the effects of graphene oxide and fullerenol C_60_(OH)_24_ nanoparticles on various subpopulations of immune system cells.

**Design of Treg differentiation study.** The study was conducted in accordance with the 2000 Declaration of Helsinki of the World Medical Association and the 1999 Protocol of the Council of Europe Convention on Human Rights and Biomedicine. Approval for the experimental protocol was obtained from the Ethics Committee of the IEGM UrBr RAS (IRB00010009) on 22 May 2024. Informed consent was obtained from each donor. The study design is presented in S2.

**Isolation and in vitro culture of regulatory T cells.** Samples of venous blood from healthy female donors (*n* = 5, age 21–40 years) were investigated. Peripheral blood mononuclear cells were obtained by density gradient centrifugation (Diacoll 1077, Dia-M, Moscow, Russia, ρ = 1.077 g/cm^3^). Monocultures of CD4^+^ cells were isolated by immunomagnetic separation using MACS^®^ MicroBeads magnetic particles (Miltenyi Biotec, Bergisch Gladbach, Germany). The purity of isolation was 95.6%. The obtained T-helper (CD4^+^) monocultures at a concentration of 10^6^ cells/mL were incubated in a 96-well plate in complete culture medium (RPMI-1640 (BioinnLabs, Rostov-on-Don, Russia) supplemented with 2 mM L-glutamine (ICN Ph., Nashville, TN, USA), 10 mM HEPES (ICN Ph., Nashville, TN, USA), 100 U penicillin, 0.1 mg/mL streptomycin, 2.5 μg/mL amphotericin B (BI, Herzliya, Israel), and 10% inactivated fetal bovine serum (BI, Herzliya, Israel)). To induce the Treg phenotype, the T lymphocyte activator “T-Cell Activation/Expansion Kit, human” (TCR activator) (Miltenyi Biotec, Bergisch Gladbach, Germany)—MACSiBead™ particles loaded with antibodies against human CD2, CD3, CD28—as well as cytokines TGF-β (5 ng/mL) and IL-2 (166 ng/mL) (Miltenyi Biotec, Bergisch Gladbach, Germany) were used. Cell cultures were incubated for 72 h at 37 °C, 5% CO_2_ in the presence of nanoparticles LP-GO and BP-GO at final concentrations of 5 and 25 μg/mL, and fullerenol C_60_(OH)_24_ at final concentrations of 25, 50, 100, and 200 μg/mL. Cultures without nanoparticle addition served as controls.

**Assessment of T-helper viability and apoptosis.** To determine viability and apoptosis, cells were stained with Zombie aqua (ZA) (Invitrogen, Carlsbad, CA, USA) and Annexin V FITC (AnnV) (BioLegend, San Diego, CA, USA). The percentage of cells in early (AnnV^+^ZA^−^) and late apoptosis/necrosis (AnnV^+^ZA^+^) was then evaluated. Analysis was performed on a CytoFlex S flow cytometer (Beckman Coulter, Brea, CA, USA). The threshold between positive (stained) and negative cell subpopulations was determined using unstained samples and fluorescence minus one (FMO) controls. Flow cytometry data were analyzed using CytExpert 2.4 software (Beckman Coulter, Brea, CA, USA).

**Figure 1:**
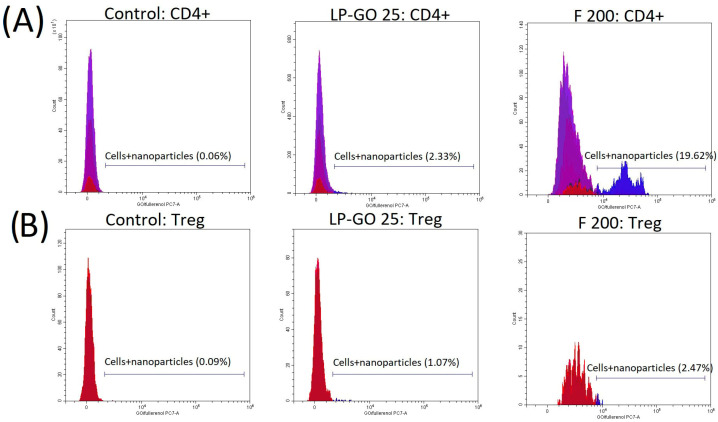
Cell gating for assessing pegylated graphene oxide nanoparticles and fullerenol C_60_(OH)_24_ sorption based on autofluorescence in the PC7 channel, exemplified by one experiment. Upper row (**A**), left to right: GO/fullerenol autofluorescence in the T-helper gate (CD4^+^) in the control culture, in the culture with 25 μg/mL LP-GO added, and with 200 μg/mL fullerenol added. Lower row (**B**), left to right: GO/fullerenol autofluorescence in the regulatory T-cell gate (CD45^+^CD4^+^CD25^+^CD127^neg/−^FOXP3^+^) in the control culture, in the culture with 25 μg/mL LP-GO added, and with 200 μg/mL fullerenol added.

**Nanoparticle sorption (adhesion/internalization).** Adhesion and internalization of fullerenol nanoparticles were assessed by measuring the autofluorescence intensity of cells in the phycoerythrin-cyanine 7 (PE-Cy7, PC7) fluorescence channel (λ_ex_ = 488 nm; bandpass filter: 750–810 nm) (Figure 1). Results are expressed as the median fluorescence intensity (MFI) in the PC7 channel of the target cell population The experiment does not distinguish between nanoparticle adhesion and internalization, so we use the term “sorption” to denote nanoparticle association on the cell surface or inside the cells.

**Assessment of Treg-Count Changes Under Nanoparticle Exposure.** The percentage of Tregs among total CD45^+^ leukocytes was assessed on a CytoFlex S flow cytometer (Beckman Coulter, Brea, CA, USA) as CD45^+^CD4^+^CD25^+^CD127^dim/−^FOXP3^+^ cells using the Treg Phenotyping Kit, anti-human, REAfinity™ (Miltenyi Biotec, Bergisch Gladbach, Germany).

Additionally, the frequencies of single-positive cells for FOXP3 and specific surface markers were determined. The proportion of CD45^+^ cells was calculated within the singlet gate, while CD25^+^, CD127^+^, and FOXP3^+^ cells were analyzed within the CD45^+^ population.

Absolute cell counts were measured via flow cytometry (cells/μL) and expressed as log_2_-transformed values.

**Assessment of IL-10 Production.** IL-10 content in cell culture supernatants was measured by enzyme-linked immunosorbent assay (ELISA) using the Interleukin-10-ELISA-BEST diagnostic kit (Vector Best, Novosibirsk, Russia) on a Multiskan Sky spectrophotometer (Thermo Fisher Scientific, Waltham, MA, USA), following the manufacturer’s instructions. Data are presented as log_2_-transformed values of cytokine concentration (pg/mL).

**Statistical Data Processing.** Statistical analysis of the data was performed in the Python 3 environment (Google Colaboratory, Mountain View, CA, USA). Normality of quantitative data distribution was tested using the Shapiro–Wilk criterion. To detect statistically significant differences, repeated measures one-way ANOVA or the nonparametric Friedman test was used in cases of deviation from normal distribution. Following significant ANOVA results, post hoc analysis with Bonferroni correction was conducted using paired *t*-tests for dependent samples (for normally distributed data) or Dunn’s multiple comparisons test (for non-normal distribution). Differences were considered statistically significant at *p* < 0.05.

## 3. Results

### 3.1. Effects of Pegylated Graphene Oxide Nanoparticles and Fullerenol C_60_(OH)_24_ on Viability of CD4+ Cells Polarized to the Treg Phenotype

**Figure 2:**
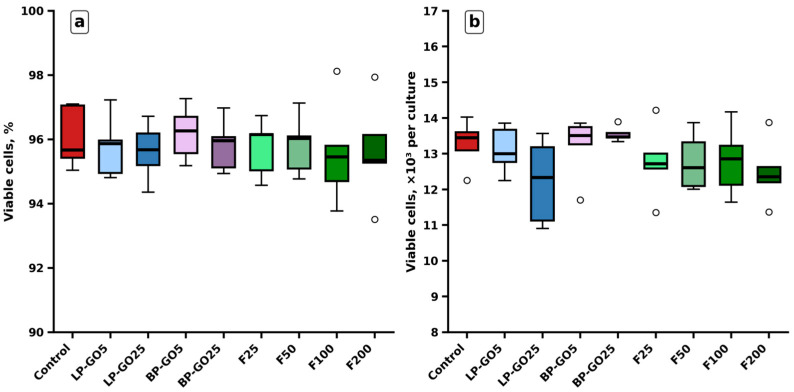
Cell viability in cultures of CD4^+^ cells polarized to the Treg phenotype during incubation with graphene oxide and fullerenol C_60_(OH)_24_ nanoparticles (median, IQR (interquartile range), outliers (circles), and *n* = 5). (**a**) percentage of viable cells; (**b**) absolute number of viable cells in the culture. X-axis: nanoparticle type and concentration (μg/mL). Shapiro–Wilk test, repeated measures one-way ANOVA, and post hoc pairwise comparisons were performed using paired *t*-tests with Bonferroni correction.

Graphene oxide nanoparticles LP-GO and BP-GO at final concentrations of 5 and 25 μg/mL did not affect T-helper (CD4^+^ cells) viability in the culture, either in terms of the percentage of live/dead cells or when calculated per cell number in the culture (Figure 2). Cell viability analysis showed that fullerenol nanoparticles across the tested concentration range (25–200 μg/mL) also exerted no statistically significant effect on T-helper viability (Figure 2). The proportion of live cells remained consistently high across all experimental groups, ranging from 95.35% to 96.15% (Figure 2a). The absolute number of viable T helpers was also consistent regardless of nanoparticle concentration, indicating no cytotoxic effects from the tested nanoparticles.

### 3.2. Effects of Pegylated Graphene Oxide Nanoparticles and Fullerenol C_60_(OH)_24_ on Apoptosis of CD4^+^ Cells Polarized to the Treg Phenotype

**Figure 3:**
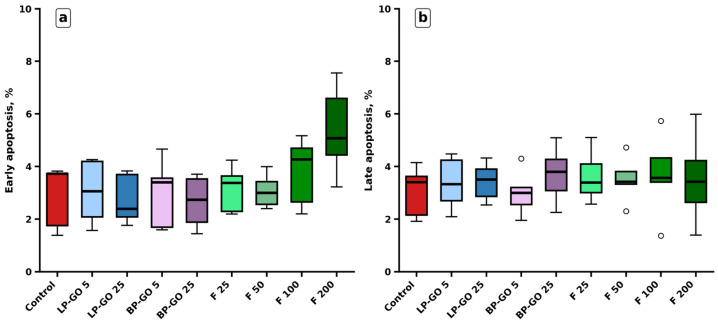
Apoptosis in cultures of CD4^+^ cells polarized to the Treg phenotype during incubation with graphene oxide and fullerenol C_60_(OH)_24_ nanoparticles (median, IQR (interquartile range), outliers (circles), and *n* = 5). (**a**) percentage of early apoptotic cells; (**b**) percentage of late apoptotic cells. X-axis: nanoparticle type and concentration (μg/mL). Shapiro–Wilk test, Friedman test, and post hoc analysis were performed using Dunn’s test with Bonferroni correction.

Assessment of apoptotic processes revealed no significant changes under the influence of graphene oxide and fullerenol C_60_(OH)_24_ nanoparticles (Figure 3). Statistical analysis showed no significant differences in markers of either early or late apoptosis between the control and experimental groups. At the same time, a visible but non-significant trend toward increased early apoptosis was observed with high fullerenol C_60_(OH)_24_ concentrations.

Overall, the data indicate that the studied nanoparticles do not exhibit pro-apoptotic activity toward T helpers.

### 3.3. T-Helper Sorption of Pegylated Graphene Oxide Nanoparticles and Fullerenol C_60_(OH)_24_

The method used in this study to assess nanoparticle sorption cannot distinguish between adhesion and internalization processes; thus, we evaluated the total nanoparticle association with cells based on the autofluorescence intensity of graphene oxide and fullerenol C_60_(OH)_24_.

**Figure 4:**
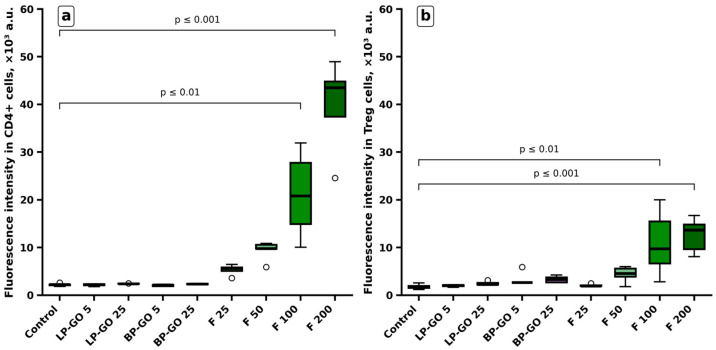
Sorption of graphene oxide and fullerenol C_60_(OH)_24_ nanoparticles by T helpers (**a**) and Tregs (**b**) during cultivation. Median, IQR (interquartile range), outliers (circles), and *n* = 5. X-axis: nanoparticle type and concentration (μg/mL). Shapiro–Wilk test, Friedman test, and post hoc analysis were performed using Dunn’s test with Bonferroni correction. Here and thereafter, *p*-values are shown only for significant differences versus the nanoparticle-free control.

Graphene oxide nanoparticles were not sorbed by T helpers or Tregs (Figure 4a,b), unlike fullerenol. At the T-helper (CD4^+^) level, fullerene C_60_(OH)_24_ sorption was significantly higher at concentrations of 100 and 200 μg/mL (Figure 4a). For Tregs (CD4^+^CD25^+^FOXP3^+^), the relative fluorescence intensity was lower than in T-helper suspensions, but fullerenol C_60_(OH)_24_ effects were similar (Figure 4b). Treg demonstrated sorption of only high fullerenol concentrations (100 and 200 μg/mL). Thus, for this process, only fullerenol concentration in the cell culture was primarily relevant. Concentrations below 100 μg/mL showed no sorption by T helpers or Tregs.

Fullerenol nanoparticles in high concentrations can, thus, form associations with human T lymphocytes, whereas PEGylated graphene oxide nanoparticles are not sorbed by these cells.

### 3.4. Effects of Pegylated Graphene Oxide Nanoparticles and Fullerenol C_60_(OH)_24_ on Treg Differentiation

**Figure 5:**
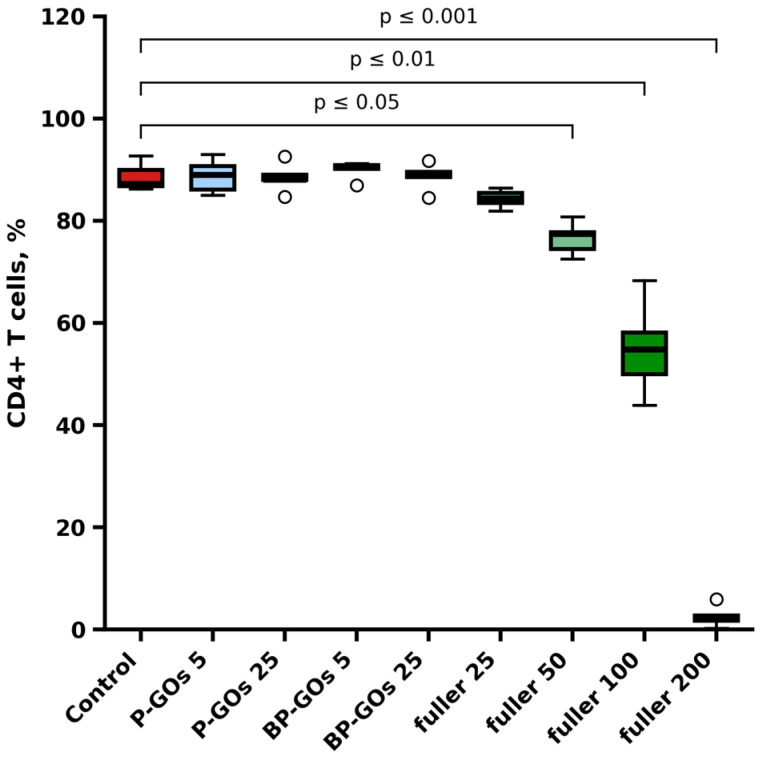
Percentage of T helpers (CD4^+^) in cell cultures polarized to the Treg phenotype during incubation with nanoparticles, %. Median, IQR (interquartile range), outliers (circles), and *n* = 5. X-axis: nanoparticle type and concentration (μg/mL) and Y-axis: the percentage of CD4+ cells within the CD45^+^ singlet gate. Shapiro–Wilk test, repeated measures one-way ANOVA, and post hoc pairwise comparisons were performed using paired *t*-tests with Bonferroni correction.

The percentage of CD4^+^ cells in the presence of graphene oxide nanoparticles LP-GO and BP-GO at final concentrations of 5 and 25 μg/mL remained unchanged, whereas in the presence of fullerenol C_60_(OH)_24_, at concentrations of 50, 100 and 200 μg/mL, it decreased statistically significantly compared to the control—from 88.5% in the control to 77.4%, 54.8% and 2.09%, accordingly (Figure 5).

Notably, interpreting this effect is challenging at present as it could represent true suppression of T-helper numbers in the culture (which would reduce well cell counts and viability) or fullerenol nanoparticles interfering with anti-CD4 antibodies on cell surfaces. This issue will be the subject of our further studies.

**Figure 6:**
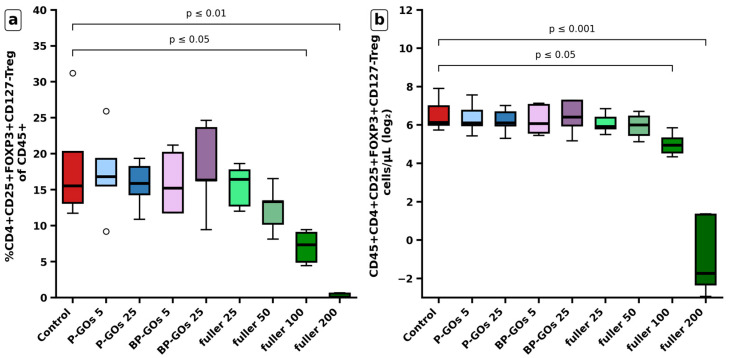
Percentage (**a**) and absolute cell count per μL (**b**) of Tregs (CD4^+^CD25^+^CD127^dim/−^FOXP3^+^) in T-helper cultures polarized to the Treg phenotype during incubation with nanoparticles, %. Median, IQR (interquartile range), outliers (circles), and *n* = 5. X-axis: nanoparticle type and concentration (μg/mL). Shapiro–Wilk test, Friedman test, and post hoc analysis were performed using Dunn’s test with Bonferroni correction.

Graphene oxide nanoparticles LP-GO and BP-GO, at concentrations of 5 and 25 μg/mL, did not alter the differentiation rate of Tregs (Figure 6). However, treatment with fullerenol C_60_(OH)_24_ resulted in a statistically significant decrease in the percentage and absolute number of Tregs, but only at 100 and 200 μg/mL concentrations (Figure 6).

A fullerenol C_60_(OH)_24_ concentration of 50 μg/mL reduced the number of CD4-expressing cells, but had no inhibitory effect on the number of Treg-phenotype cells.

Individual assessment of marker expression, including FOXP3, revealed no change in the percentage of CD45^+^ and CD25^+^ cells in the cultures. Their absolute counts were reduced by 200 μg/mL fullerenol C_60_(OH)_24_. However, the percentage and absolute count of CD127^+^ cells increased after treatment with 100 and 200 μg/mL fullerenol, whereas the percentage and absolute count of FOXP3^+^ cells decreased 10- and 9-fold, respectively (Supplementary S3).

Since the cultures consisted of pre-isolated CD4^+^ cells, we omitted this marker from the gating sequence to avoid potential fullerenol C_60_(OH)_24_ nanoparticle interference with antibody fluorescence or non-specific effects on CD4 molecules. An alternative gating strategy for Tregs, omitting the CD4 marker, also demonstrated a reduction in both the percentage and absolute count of CD45^+^CD25^+^CD127^dim/−^ cells following treatment with the two highest concentrations of fullerenol C_60_(OH)_24_ (Supplementary S3).

Thus, concentrations of up to 25 μg/mL of all nanoparticles, including fullerenol C_60_(OH)_24_, had no effect on Treg differentiation. Higher concentrations of fullerenol C_60_(OH)_24_ inhibited regulatory T-cell differentiation.

### 3.5. Effects of Pegylated Graphene Oxide and Fullerenol C_60_(OH)_24_ Nanoparticles on IL-10 Production by Tregs

**Figure 7:**
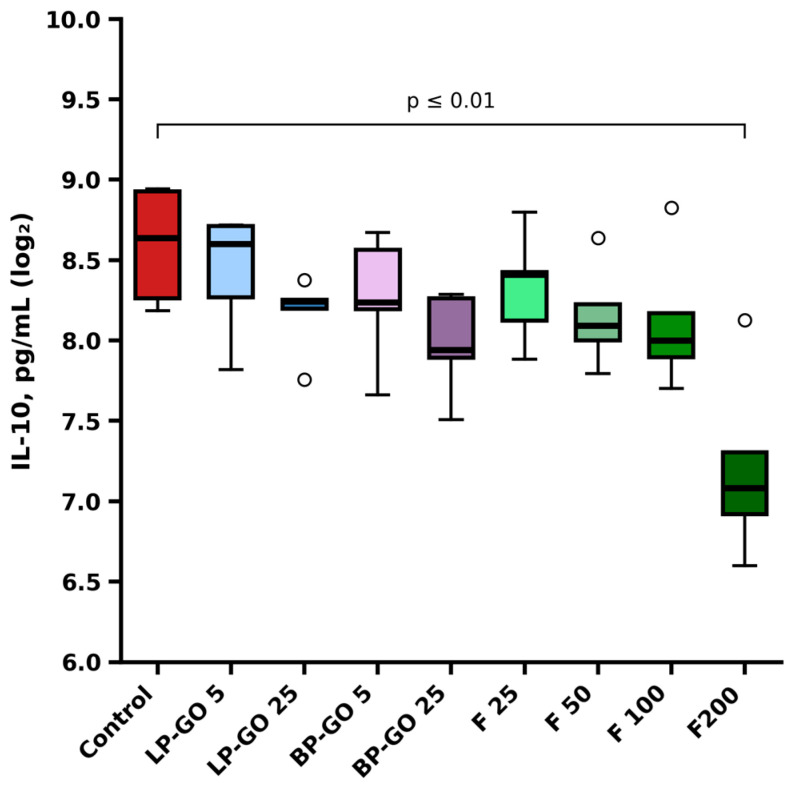
Effects of fullerenol C_60_(OH)_24_ nanoparticles on IL-10 production by T helpers induced to the Treg phenotype under in vitro conditions. X-axis: nanoparticle type and concentration (μg/mL) and Y-axis: log_2_ of IL-10 concentration (pg/mL) (mean ± SD, *n* = 5). Shapiro–Wilk test, Friedman test, and post hoc analysis were performed using Dunn’s test with Bonferroni correction. Outliers are marked as circles.

LP-GO, BP-GO, and fullerenol nanoparticles up to 100 µg/mL showed no statistically significant effect on IL-10 production in T-helper cultures polarized to the Treg phenotype, regardless of concentration. However, fullerenol C_60_(OH)_24_ at a concentration of 200 µg/mL exerted an inhibitory effect on the production of this cytokine (Figure 7).

## 4. Discussion

Analysis of the literature on carbon nanoparticle (CNP) interactions with Tregs reveals that ex vivo studies demonstrate direct effects that vary by functionalization [11]. For instance, GITR-targeted CNPs enhance Treg capture in immunotherapy, though the mechanisms of dysfunction remain unclear [11].

PEG-coated single-walled carbon nanotubes loaded with GITR ligands (SWCNT-PEG-GITR) enable selective internalization by Tregs in the tumor microenvironment via receptor-mediated endocytosis, translocating to the cytoplasm and nucleus ex vivo and in vivo, thereby impairing Treg function [11].

In our study, flow cytometry and nanoparticle autofluorescence were used to assess sorption. Previously, similar graphene oxide derivatives modulated Th17 differentiation in vitro depending on their functionalization; specifically, LP-GO (25 µg/mL) boosted Th17 and Th22 cell populations as well as IFN-γ production, while BP-GO (25 µg/mL) reduced classical Th17 cells and IL-17 levels [27].

Graphene oxide showed no adsorption or internalization by T helpers or Tregs, which contrasts with the findings of Ding et al. [30], who reported that unmodified and carboxylated GO (GO-COOH) sorbed on T lymphocyte surfaces, while PEI-functionalized GO (GO-PEI) penetrated the nuclei via membrane damage.

According to our previous study, fullerenol C_60_(OH)_24_ exhibited mild cytotoxicity toward T cells only at 100–200 µg/mL (a 1–2.5% decrease in viability), whereas B cells remained resistant; furthermore, time- and dose-dependent sorption was higher in B cells than in T cells [29].

In the present study, although we did not observe a decrease in T-helper-cell viability upon exposure to any of the nanoparticle types or concentrations tested, fullerenol C_60_(OH)_24_ at 100 and 200 µg/mL caused a noticeable trend toward an increased percentage of apoptotic cells. Cells in the early apoptotic stage are capable of losing surface molecules, which likely explains the dramatic decrease in CD4 expression observed in our study [31]. Analysis of other surface markers revealed that T-helper-cell differentiation was impaired, despite the absence of a pronounced cytotoxic effect. Crucially, a decrease in the concentration of the cytokine IL-10 produced by Tregs confirms this inhibitory effect. These data are consistent with our previous findings regarding the effects of PEGylated graphene oxide nanoparticles on Th17 differentiation [27].

At pharmacological concentrations (5–50 μg/mL), none of the nanoparticle types significantly affected the viability or differentiation of T helpers into Tregs, confirming the potential of these nanomaterials for use in the development of nanobiomedical products. However, our findings highlight the importance of further investigating the effects of non-cytotoxic concentrations of nanoparticles on immune cell functions.

For comparison, PLGA-based aAPCs loaded with IL-2 and TGF-β and coated with anti-CD3/CD28 induced CD4^+^CD25^hi^FOXP3^+^CD127^−^ and CD8^+^FOXP3^+^ Tregs in vitro [32]. In another study, carbosilane dendrimers preserved Treg viability and phenotype during HIV-1 challenge without disrupting FOXP3 expression.

Although nanotechnology enables precise Treg targeting, the field still faces significant challenges, such as the low abundance of these cells and a limited number of specific cellular targets. Importantly, while the treatment of autoimmunity and transplant rejection favors Treg induction, tumor therapies require their suppression. Therefore, successful clinical translation demands comprehensive assessments of long-term toxicity, nanoparticle stability, and further in vivo studies.

## 5. Conclusions

Overall, we demonstrated that graphene oxide nanoparticles LP-GO and BP-GO at concentrations of 5 and 25 μg/mL, and fullerenol C_60_(OH)_24_ at concentrations ranging from 50 to 200 μg/mL, exerted no significant effects on T-helper viability or early and late apoptosis parameters (Table 2). Cell viability remained consistently high across all experimental groups, ranging from 95.35% to 96.15%, indicating no cytotoxic effects from the studied nanoparticles.

Sorption analysis at the T-helper (CD4^+^) and Treg (CD4^+^CD25^+^FOXP3^+^) levels revealed that only high fullerenol C_60_(OH)_24_ concentrations (100 and 200 μg/mL) showed association with these cells. In cultures with lower concentrations of fullerenol, it was not sorbed by T-helper or Treg cells, as were graphene oxide nanoparticles LP-GO and BP-GO (Table 2).

The percentage of CD4^+^ cells significantly decreased in the presence of fullerenol C_60_(OH)_24_ at 50, 100 and 200 μg/mL compared to the control. At the Treg level, graphene oxide nanoparticles LP-GO and BP-GO had no significant effect on the number of cells in the culture. Fullerenol C_60_(OH)_24_ significantly decreased Treg numbers at concentrations of 100 and 200 μg/mL. In addition, 200 μg/mL of fullerenol reduced the culture levels of IL-10.

As previously noted in the introduction, T helper cells and Tregs exhibit metabolic differences that may determine their differential sensitivity to fullerenol C_60_(OH)_24_. It is hypothesized that T helpers are more sensitive to fullerenol C_60_(OH)_24_ exposure due to their more active metabolism. Conversely, Tregs appear more resistant owing to their lower glycolysis rate, which apparently affects membrane fluidity [33], resulting in less active sorption of fullerenol C_60_(OH)_24_ nanoparticles. This fact may explain the decrease in T helpers, but not T reg, under the influence of 50 μg/mL fullerenol C_60_(OH)_24_.

Thus, we must emphasize that the absence of cytotoxicity of the nanomaterial does not mean that there is no effect on cell differentiation and function.

**Limitations**: The sorption of nanoparticles (adhesion/internalization) was assessed using only one method, based on flow cytometry data for the autofluorescence of GO and fullerenol nanoparticles in the PC7 channel. This method does not allow for distinguishing between nanoparticle adhesion and internalization. Additionally, we had no opportunity to evaluate the potential masking effect, where the presence of nanoparticles prevents detection of molecules on the cell surface.

## Figures and Tables

**Table 1:** Characteristics of the pegylated graphene oxide and fullerenol C_60_(OH)_24_ nanoparticles used.

	LP-GO	BP-GO	Fullerenol C_60_(OH)_24_
Diameter, nm	184 ± 73	287 ± 52	186 (DLS, in water) 356 ± 167 (TEM, in water) 308 ± 110 (TEM, in the complete culture medium)
Polydispersity index	0.25 ± 0.02	0.23 ± 0.02	0.45 (DLS, in water)
Zeta potential, mV	−31.70 ± 1.70	−34.28 ± 0.41	−26.0 ± 6.31
PEG mass fraction, %	17.2 ± 1.4	20.5 ± 1.8	—

Note: data are presented as mean ± SD.

**Table 2:** Investigation of the direct effects of graphene oxide and fullerenol C_60_(OH)_24_ nanoparticles on regulatory T cells in vitro.

Parameters	Graphene Oxide Nanoparticles LP-GO and BP-GO 5 and 25 μg/mL	Fullerenol C_60_(OH)_24_
T-helper viability, %	↔	↔
T-helper viability, abs.	↔	↔
Early apoptosis, %	↔	↔
Late apoptosis, %	↔	↔
Sorption, T-helpers	↔	100, 200 μg/mL ↑
Sorption, Treg	↔	100, 200 μg/mL ↑
Treg, %	↔	100, 200 μg/mL ↓
T-helpers, %	↔	50, 100, 200 μg/mL ↓
IL-10, pg/mL,	↔	100, 200 μg/mL ↓

Note: ↑—increase; ↓—decrease; ↔—no effect.

## Data Availability

Datasets are available from the corresponding author on reasonable request. AI tools (Perplexity AI, Inc., San Francisco, CA, USA) were used to assist with translation of the manuscript. The authors reviewed, edited, and verified the final text and take full responsibility for its content.

## Supplementary Materials

The following supporting information can be downloaded at: https://www.mdpi.com/article/10.3390/nano16110667/s1, S1: Study Design; S2: Nanoparticles Characterization, S3: additional flow cytometry results.

## Abbreviations

The following abbreviations are used in this manuscript:

BP-GO	Branched polyethylene glycol-coated graphene oxide nanoparticles
CNP	Carbon nanoparticles
CTLA-4	Cytotoxic T-lymphocyte–associated protein 4
GITR	Glucocorticoid-induced TNFR-related protein
GO	Graphene oxide
IL	Interleukin
LP-GO	Linear polyethylene glycol-coated graphene oxide nanoparticles
PEG	Polyethylene glycol
TGF-β	Transforming growth factor β
Th	T helper cell
Treg	Regulatory T cell
