# Supplementary material for: A Study of the Direct Effect of Pegylated Graphene Oxide Nanoparticles and Fullerenol C60(OH)24 on the Differentiation of Regulatory T Cells In Vitro
Source: Nanomaterials (Basel). 2026 May 26;16(11):667. doi: 10.3390/nano16110667 (PMC13257590; doi:10.3390/nano16110667)
Supplement: Supplementary file 1 [file nanomaterials-16-00667-s001.zip › Supplementary S2.pdf]

Supplementary S2. Nanoparticles caracterization

GRAPHENE OXIDE CHARACTERIZATION

Methods

Graphene oxide powder (lateral size of 100–200 nm) was obtained from Ossila Ltd. (Sheffield, UK). Monochloroacetic acid, 1-(3-dimethylaminopropyl)-3-ethylcarbodiimide hydrochloride (EDC), N-hydroxysuccinimide (NHS), 8-arm-PEG-NH_2 (10,000 kDa), methoxy polyethylene glycol amine (5 kDa) were obtained from Alfa Aesar (Ward Hill, MA, USA). Solutions were prepared with deionized water. All reagents were used without additional purification.

Instrumentation. IFS 66/S IR spectrometer and Raman microscope SENTERRA were obtained from Bruker Corporation (Billerica, MA, USA). UV 2600 two-beam spectrophotometer was obtained from Shimadzu (Kyoto, Japan). ZetaPALS was obtained from Brookhaven Instruments Corporation (Wakefield, MA, USA). TGA/DSC 1 combined with TG-DSC device was obtained from Mettler-Toledo (Greifensee, Switzerland).

Carbodiimide-mediated coupling of PEG-NH_2 and GO was made as follows. Aqueous solutions of GO (2 mg/mL) were sonicated for 30 min using probe sonicator (output power was 25 W), and then carboxylated by the addition of Cl-CH_2-COOH (18.5 mM) under sonication for 60 min. Carboxylated GO nanoparticles were washed by centrifugation at 10,000 g with water until neutral pH level was reached. EDC (to 4 mM), NHS (to 10 mM), and branched or linear PEG (to 2 mg/mL) were added to the suspension of carboxylated GO (pH 5.6) over the course of 5 min with constant ultrasonic treatment. The suspension was kept for 24 h at room temperature, GO-PEG was purified by dialysis, washed three times with ethanol by centrifugation (10,000 g), and dried under vacuum at +65 °C.

GO coated with linear and branched PEG will be further referred to as LP-GO and BP-GO, respectively. Designation "GO-PEG" will be used as a generic term for any type of PEGylated graphene oxide.

FTIR spectra of the intact and PEGylated GO were obtained in the range 400–4000 cm−1 using KBr tablets (2 mg of nanoparticles per 299 mg of KBr). UV-Vis spectra of GO-PEG were recorded in the range of 200–900 nm. Hydrodynamic diameter and zeta potential were measured by dynamic light scattering (DLS). The percentage of the attached polymer was quantified by thermogravimetric analysis (TGA) at a heating rate of 10 K min−1 from 30 to 900 °C in an inert atmosphere. Raman spectra (D and G bands intensities) were obtained in the range 500–2000 cm−1 (laser power: 0.2 mW; wavelength: 532 nm). Scanning electron microscopy (SEM) images were obtained under an acceleration voltage of 20 kV.

Results

GO nanoparticles with lateral sizes of 100–200 nm were functionalized with PEG, a polymer capable of improving colloidal stability and the toxicity profile of nanomaterials. Two types of PEG were used for nanoparticle coating, namely linear PEG and branched PEG (8-armed PEG).

Pristine GO nanoplatelets were treated with chloroacetic acid to introduce additional carboxylic groups. Then, aminated PEG was covalently attached to carboxylated GO via carbodiimide chemistry. The resulting nanoparticles were characterized by various techniques including FTIR, Raman spectroscopy, elemental analysis (EDS), TGA, SEM, and DLS (Figure S1).

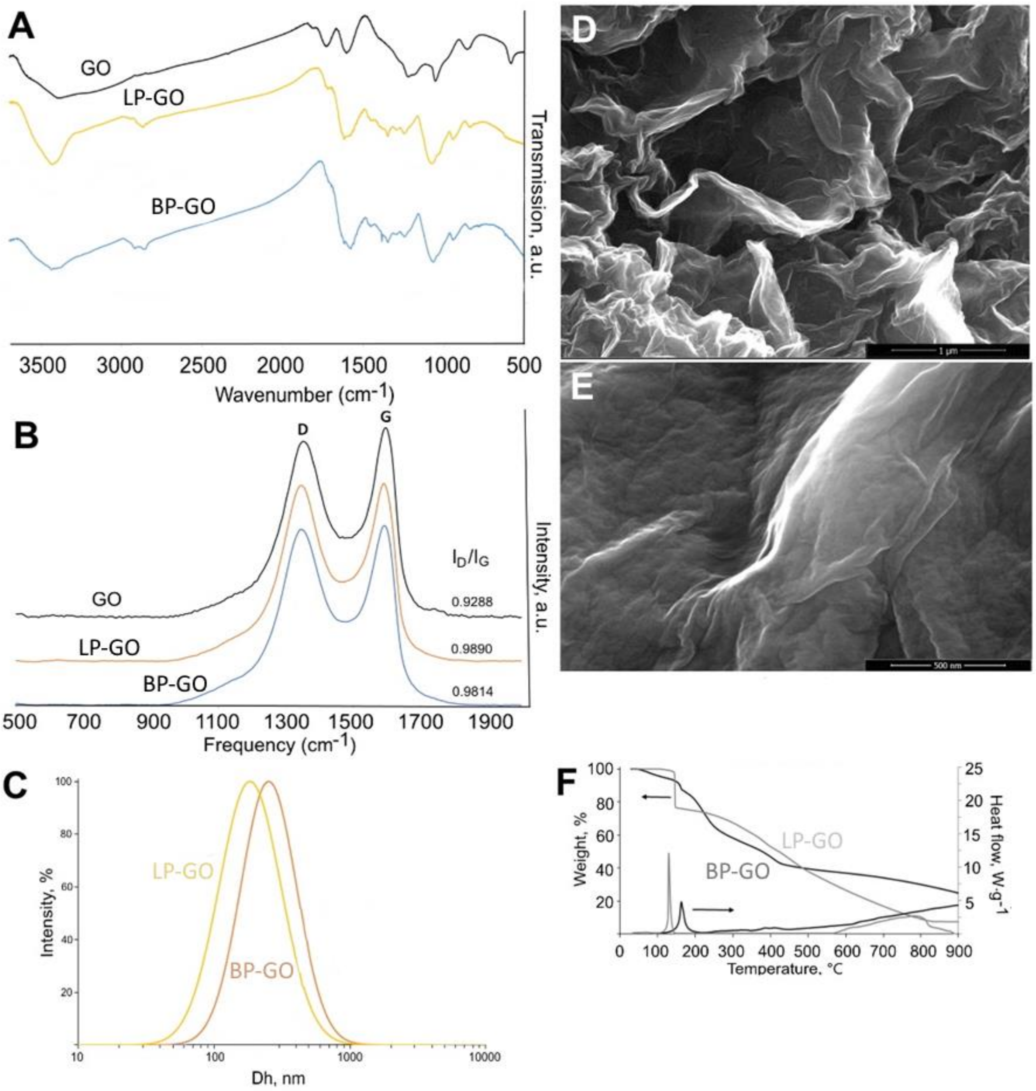

Figure S1. Characterization of GO-PEG. (A)—FTIR spectra; (B)—Raman spectra; (C)—intensity-weighted size distribution determined by DLS; (D,E)—SEM images of GO (D) and GO-PEG (E); (F,G)—TGA/DSC of GO-PEG. Scale bars are 1 μm (D) and 500 nm (E).

Typical intense absorption bands (a.b.) at 3400 cm^{-1} (-OH), 1720 cm^{-1} (C=O), 1600 cm^{-1} (C=C), 1220 cm^{-1} (C-O), and 1070 cm^{-1} (C-O-C) were observed in the IR spectrum of the pristine graphene oxide, evidencing the presence of carbonyl, carboxyl, alkoxy, epoxy and hydroxyl groups in the sample. Following the pegylation one could observe both changes in the intensity of the pre-existing absorption bands and the emergence of novel typical absorption bands. Specifically, a.b. at 2870 cm^{-1} ($-CH_2-$) and 1640 cm^{-1} (-NH-CO-) are detected and the a.b. intensity at 1070 cm^{-1} (C-O-C) is significantly elevated, indicating the presence of PEG in the system as well as for the amide bond formation between amine groups of PEG and GO. One should note the weakening in the a.b. intensity at 1420 cm^{-1} (C-OH) that additionally supports the amide bond formation between -COOH groups of GO and $-NH_2$ groups of $PEG-NH_2$.

Using Raman scattering spectroscopy it was found that GO PEGylation was manifested in an increase in oxygen amount (ID/IG increases from 0.93 to 0.98) and decrease in the proportion of carbon moieties with sp2 bonds. Values obtained on the relationships of typical band intensities (ID/IG) could be interpreted as showing that the processes of carboxylation and subsequent PEGylation did not destroy the aromatic structure of monolayered graphene oxide.

Hydrodynamic diameters and polydispersity indices of GO-PEG are presented in **Table S1**. DLS technique is based on the Einstein–Stokes equation describing the behavior of spherical particles. The zeta potential of GO-PEG is lower than −30 mV, that facilitates their good colloidal stability upon storage in deionized water.

Table S1. Properties of GO-PEG.

	LP-GO	BP-GO
Dh, nm	184 ± 73	287 ± 52
PdI	0.25 ± 0.02	0.23 ± 0.02
Zeta Potential, mV	−31.70 ± 1.70	−34.28 ± 0.41
PEG Coverage, wt%	17.2 ± 1.4	20.5 ± 1.8

Dh—hydrodynamic diameter, PdI—polydispersity index.

It is known that GO loses weight in an inert atmosphere at 150–300 °C due to the thermal decomposition of oxygen-containing groups. This thermal decomposition is accompanied by an exothermic effect. For the studied samples, BP-GO in a narrow temperature range of 150–170 °C, sharp decreases in mass are observed (Figure S1 F), which is probably associated with the transformation of the various oxygen-containing GO groups (carbonyl, carboxyl, alkoxy, epoxy, and hydroxyl groups). For PEGylated

samples, one more stage of weight loss is observed in the temperature range 250–450 °C, caused by thermal decomposition of the main chains of the branched PEG polymer. Calculations based on TGA data showed that BP-GO contain about 20 wt% of PEG (Table S1).

The results obtained correspond to those for the samples of GO coated with linear PEG, with the only difference being that the intensities of thermal effects differ and the weight drops in the first temperature range of 150–300 °C are not so pronounced. The character of the TGA dependences for non-coated graphene oxide is similar to the literature data. However, the data obtained indicate a high oxidation state of the GO nanoparticles (about 58 wt% of oxygen). Calculations based on TGA data showed that the LP-GO samples contain about 17–19 wt% of PEG (Table S1).

Figure S1 shows typical scanning electron micrographs of the intact GO and GO-PEG. The rougher surface of PEGylated GO indicates the presence of polymer.

The presence of the PEG polymer on the GO surface was confirmed by elemental analysis using energy-dispersive X-ray spectroscopy (EDAX), implemented on a scanning electron microscope. Elemental analysis was performed on the intact GO and BP-GO. Examples of mapping and spectra are shown in **Figure S2**. Intact GO is characterized by the presence of only carbon and oxygen in a ratio of 85:15 at%. In the BP-GO sample, in addition to carbon and oxygen, the presence of nitrogen was observed. The atomic ratio of elements in the sample was changed to 74:2:24 (C:O:N). These changes are most likely also associated with the appearance of the branched PEG on the surface of GO nanoparticles.

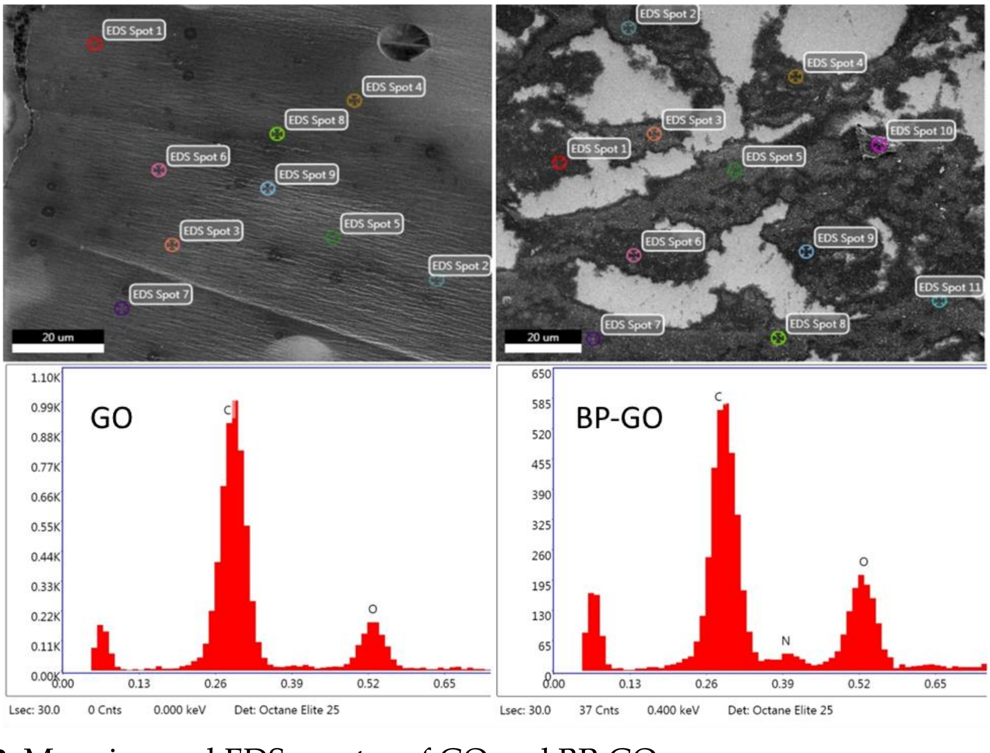

Figure S2. Mapping and EDS spectra of GO and BP-GO.

FULLERENOL $C_{60}(OH)_{24}$ CHARACTERIZATION

Methods

The study utilized fullerenol $C_{60}(OH)_{22–24}$ (MST-WS60-Bio, Modern Synthesis Technology, St. Petersburg, Russia). Since the content of $C_{60}(OH)_{22}$ declared by the manufacturer in the fullerenol $C_{60}(OH)_{22–24}$ preparation was ≈ 0.01%, the fullerenol is designated only by the formula $C_{60}(OH)_{24}$.

Fullerenol was dissolved in distilled water and diluted to the final concentration of 200 μg/mL. The sample was treated with ultrasound for 30 min at a 70% power setting. Nanoparticle size was measured using dynamic light scattering (DLS) with BeNano 180 Zeta Pro (Bettersize, Dandong, China) in a polystyrene cuvette at room temperature. The measurement angle was set to 90°. Volume-based particle size distributions were obtained and the median values of three measurements were calculated. The size value exhibiting the highest frequency is reported. The zeta-potential of a 100 μg/mL solution of fullerenol in distilled water was measured using BeNano 180 Zeta Pro (China) in a polystyrene cuvette at room temperature. Six measurements were performed; mean value and standard deviation are reported.

1 μL of fullerenol suspension was deposited onto a 3 mm copper grid with Formvar/Carbon support film (TedPella, Inc., Redding, CA, USA). The dried specimen was examined using a Hitachi HT7700 Exalens transmission electron microscope (Hitachi, Tokyo, Japan) operating in high-resolution (HR) mode at a 100 kV acceleration voltage and 15 μA current.

Fullerenol at 5 μg/mL in water and at 50 μg/mL in a complete culture medium was used and images were taken at a magnification of 30,000. Acquired images in TIFF format were analyzed using Fiji (ImageJ 1.54p) to measure particle diameter in nm. The microscope was calibrated to 1.974 nm/pixel at 10,000 magnification prior to imaging. The particle diameter was measured manually using ImageJ and the measurement results were exported and then analyzed.

The IR spectrum of fullerenol was recorded using a Bruker Vertex 80V FTIR spectrometer (Bruker, Billerica, MA, USA). The sample was ground in an agate mortar and pressed into a pellet with KBr additive at a ratio of 1:300 for transmission measurements. The spectrum was acquired by averaging 16 scans at a resolution of 2 cm^{-1} over the wavelength range of 400–4000 cm^{-1}.

The trace element content was determined using inductively coupled plasma mass spectrometry (ICP-MS) with a Bruker AURORA M90 spectrometer (USA). Element concentrations in the samples were calculated using the spectrometer firmware. The accuracy of the measurements was verified using the reference standard "Trace Metals in Drinking Water" from High-Purity Standards (Charleston, VA, USA). Laboratory analyses were carried out at the Shared Research Facilities Center of Perm State National Research University.

The fluorescence spectrum was measured using a Synergy H1 hybrid reader (BioTek Instruments, Winooski, VT, USA) at 37 °C. Measurements were carried out across an excitation wavelength (λ_{exc}) range of 270 nm to 650 nm with 20 nm increments. Fluorescence intensity was recorded in the range of λ_{exc} + 30 nm up to 700

nm. For the measurement, 200 μL of an aqueous solution of fullerenol $C_{60}(OH)_{24}$ (200 μg/mL) was added to a well of a black 96-well polystyrene microplate.

Results

Our data on the absorbance spectrum of fullerenol $C_{60}(OH)_{24}$ in the UV-Vis range are presented in **Figure S3**. Fullerenol solution absorbs light in the broad spectrum of 200–600 nm with the absorbance value monotonously decreasing. At the same time no absorbance in the red part of the spectrum was observed.

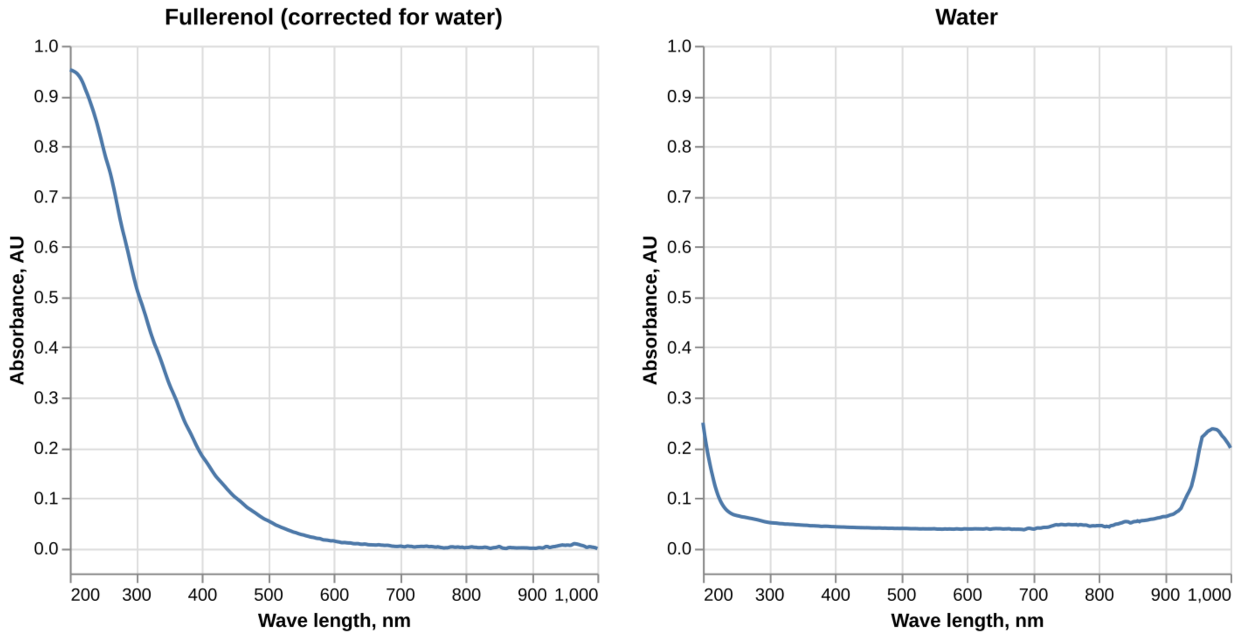

Figure S3. UV-Vis absorbance spectrum of fullerenol $C_{60}(OH)_{24}$ solution in water.

Fullerenol $C_{60}(OH)_{24}$ demonstrates a broad fluorescence spectrum, with the highest intensity of fluorescence occurring at the λ_{ex} = 430 nm and an emission maximum at around 560 nm (**Figure S4**).

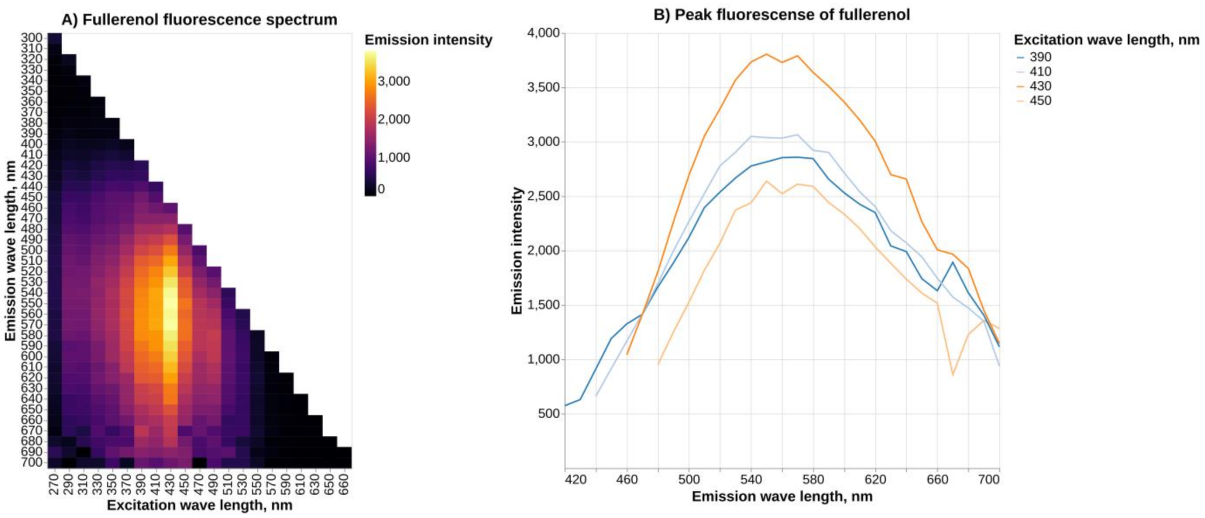

Figure S4. Fluorescence spectrum of fullerenol $C_{60}(OH)_{24}$ in water.

Analysis of metal impurities using ICM-MS determined the presence of sodium in the fullerenol sample (**Figure S5**). At the same time the concentrations of other metal elements remained at the background level.

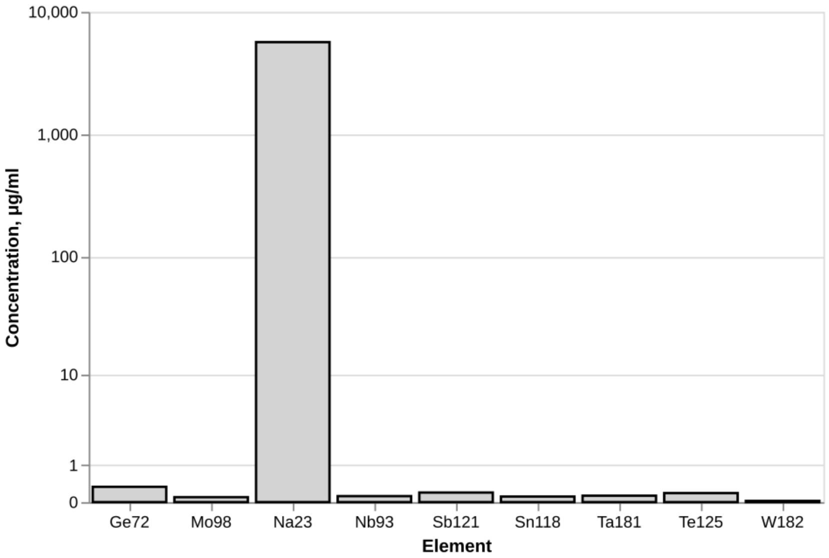

Figure S5. Metal impurities in fullerenol $C_{60}(OH)_{24}$.

A linear calibration curve for the determination of endotoxin content was obtained with the R^2 value of 0.982. Fullerenol concentration in the sample containing 10 μg/mL of nanoparticles was measured to be 0.012 EU/mL, which corresponds to the endotoxin content of 1.2×10^{-3} EU/μg for dry fullerenol.

The IR spectrum exhibits the following characteristic absorption bands (**Figure S6**): 3424 cm^{-1} (O–H stretching vibrations), 1595 cm^{-1} (C=C bending vibrations), 1390 cm^{-1} (bending vibrations of the C–O–H functional group), and 1060 cm^{-1} (stretching vibrations of the C–O functional group).

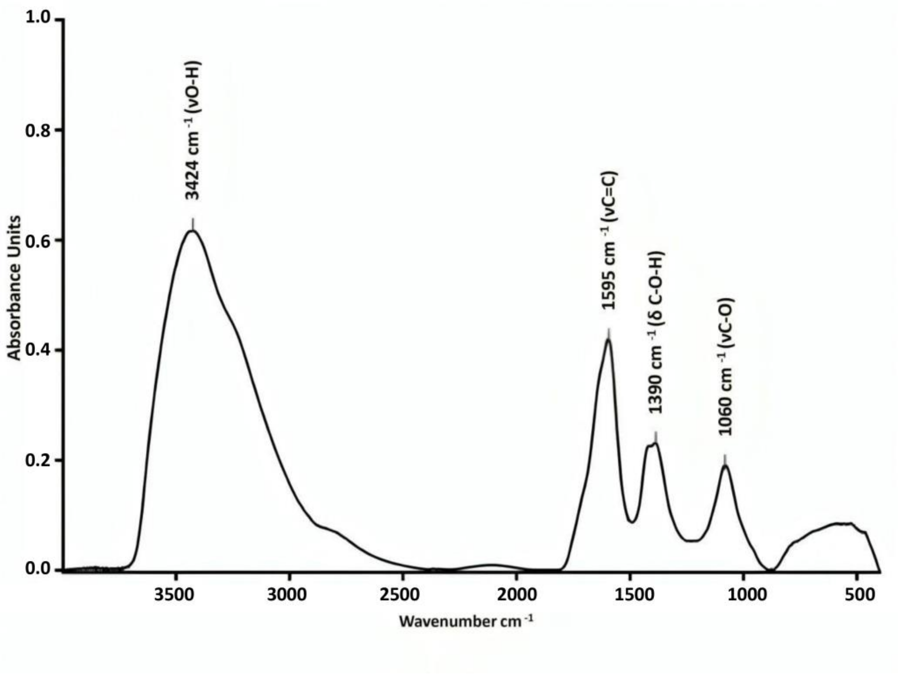

Figure S6. IR spectrum of fullerenol C60(OH)24.

Size measurement of fullerenol nanoparticles using DLS in water shows that the volume-based particle diameter is 186 nm with the polydispersity index (PDI) of 0.45. It can be noted from the plot (**Figure S7**) that intensity-based size distribution shows the presence of large aggregates absent in the volume-based distribution, which can be attributed to the fact that intensity-based metrics are more sensitive to the presence of large particles. The zeta-potential of a 100 μg/mL solution of fullerenol in distilled water is −26 ± 6.31 mV (mean ± SD).

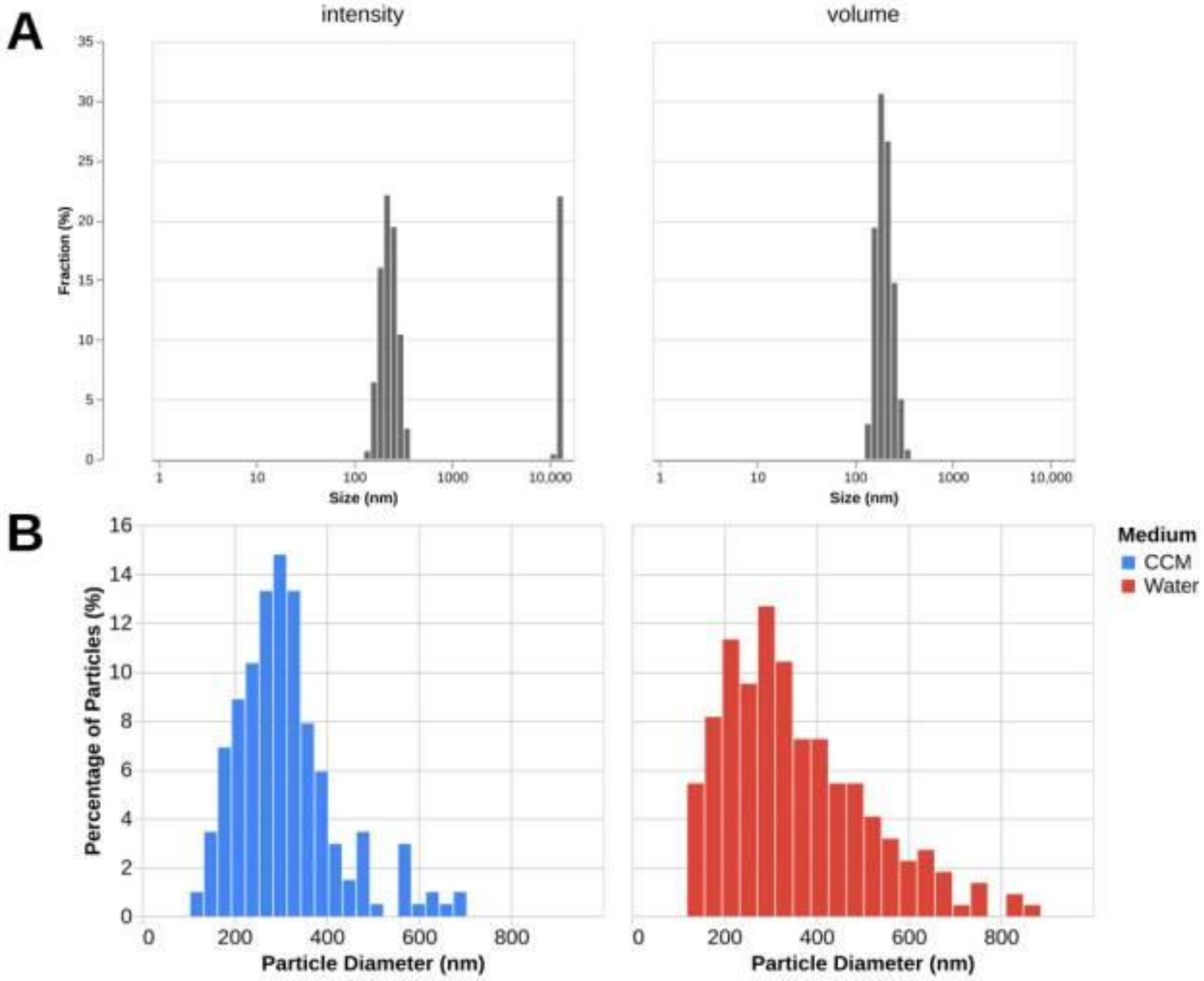

Figure S7. $C_{60}(OH)_{24}$ determined by DLS and TEM. (A)—Intensity- and volume-based distributions as measured by DLS. Mean values for 3 measurements are displayed. (B)—Particle diameter distributions measured by TEM in water (222 measurements) and complete culture medium (203 measurements).

The mean particle diameters obtained from TEM images are 356 ± 167 (mean ± SD) in water and 308 ± 110 (mean ± SD) in the complete culture medium (Figure S7). The data show a wide distribution of sizes, which is consistent with DLS. The discrepancy between the mean diameter values of the DLS and TEM measurements is expected as the methods measure different parameters (hydrodynamic diameter and core diameter) of the samples in different states (dissolved in water and dried).
