# Supplementary material for: A Study of the Direct Effect of Pegylated Graphene Oxide Nanoparticles and Fullerenol C60(OH)24 on the Differentiation of Regulatory T Cells In Vitro
Source: Nanomaterials (Basel). 2026 May 26;16(11):667. doi: 10.3390/nano16110667 (PMC13257590; doi:10.3390/nano16110667)
Supplement: Supplementary file 1 [file nanomaterials-16-00667-s001.zip › Supplementary S3.pdf]

Supplementary S3

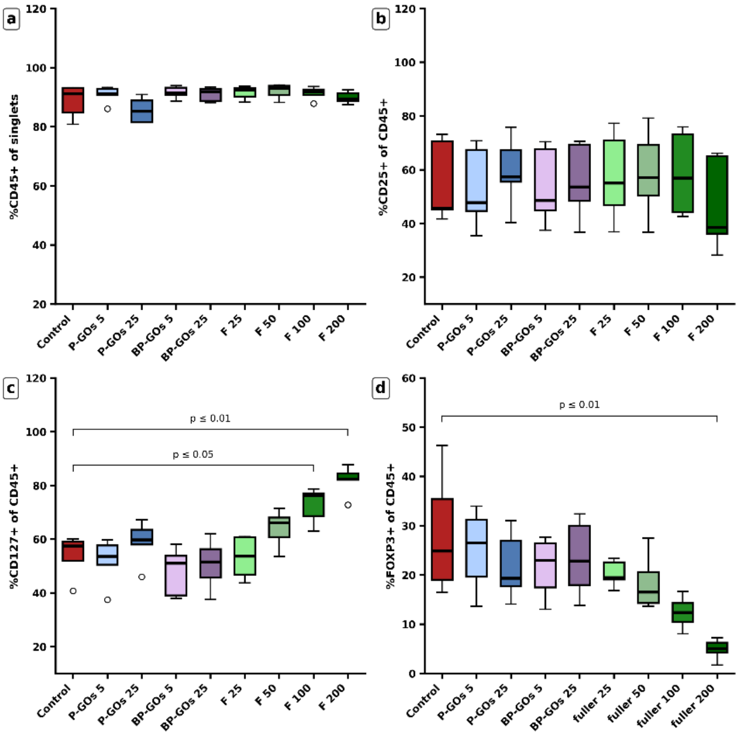

Figure S8. Percentage of (a)CD45+ (from singlets), (b)CD25+, (c)CD127+, (d)FOXP3+ (from CD45+ singlets) in CD4+ cell cultures. Median, IQR (interquartile range), outliers; n=5. X-axis: nanoparticle type and concentration (μg/mL); Y-axis: the percentage of cells. Shapiro-Wilk test, Friedman test, Post-hoc analysis was performed using Dunn's test with Bonferroni correction.

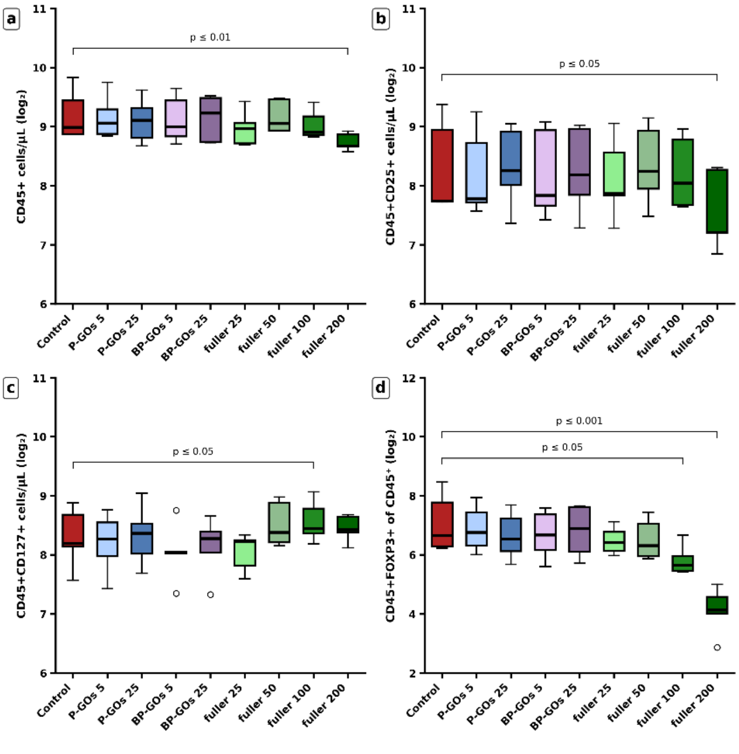

Figure S9. Absolute counts of (a)CD45+ (from singlets), (b)CD25+, (c)CD127+, (d)FOXP3+ (from CD45+ singlets) in CD4+ cell cultures. Median, IQR (interquartile range), outliers; n=5. X-axis: nanoparticle type

and concentration (µg/mL); Y-axis: absolute cell count per µL. Shapiro-Wilk test, Friedman test, Post-hoc analysis was performed using Dunn's test with Bonferroni correction.

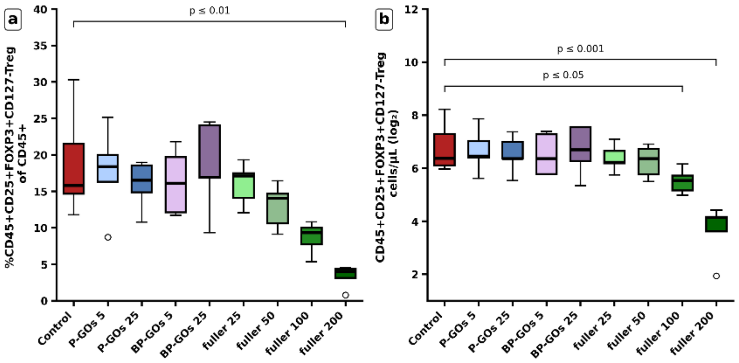

Figure S10. Percentage (a) and absolute cell count per 1 µL (b) of Tregs (CD45+CD25+ FOXP3+CD127dim/-) in T helper cultures polarized to the Treg phenotype during incubation with nanoparticles. Median, IQR (interquartile range), outliers; n=5. X-axis: nanoparticle type and concentration (µg/mL); Y-axis: a - percentage of CD25+FOXP3+CD127dim/- cells in CD45+ cell gate; log2 of absolute cell count per 1 µL. Shapiro-Wilk test, Friedman test, Post-hoc analysis was performed using Dunn's test with Bonferroni correction.
