# Supplementary figures and images for: A Study of the Direct Effect of Pegylated Graphene Oxide Nanoparticles and Fullerenol C60(OH)24 on the Differentiation of Regulatory T Cells In Vitro
Source: Nanomaterials (Basel). 2026 May 26;16(11):667. doi: 10.3390/nano16110667 (PMC13257590; doi:10.3390/nano16110667)

## Supplementary S1. Study Design

### STUDY DESIGN [*in vitro* Activation Model]

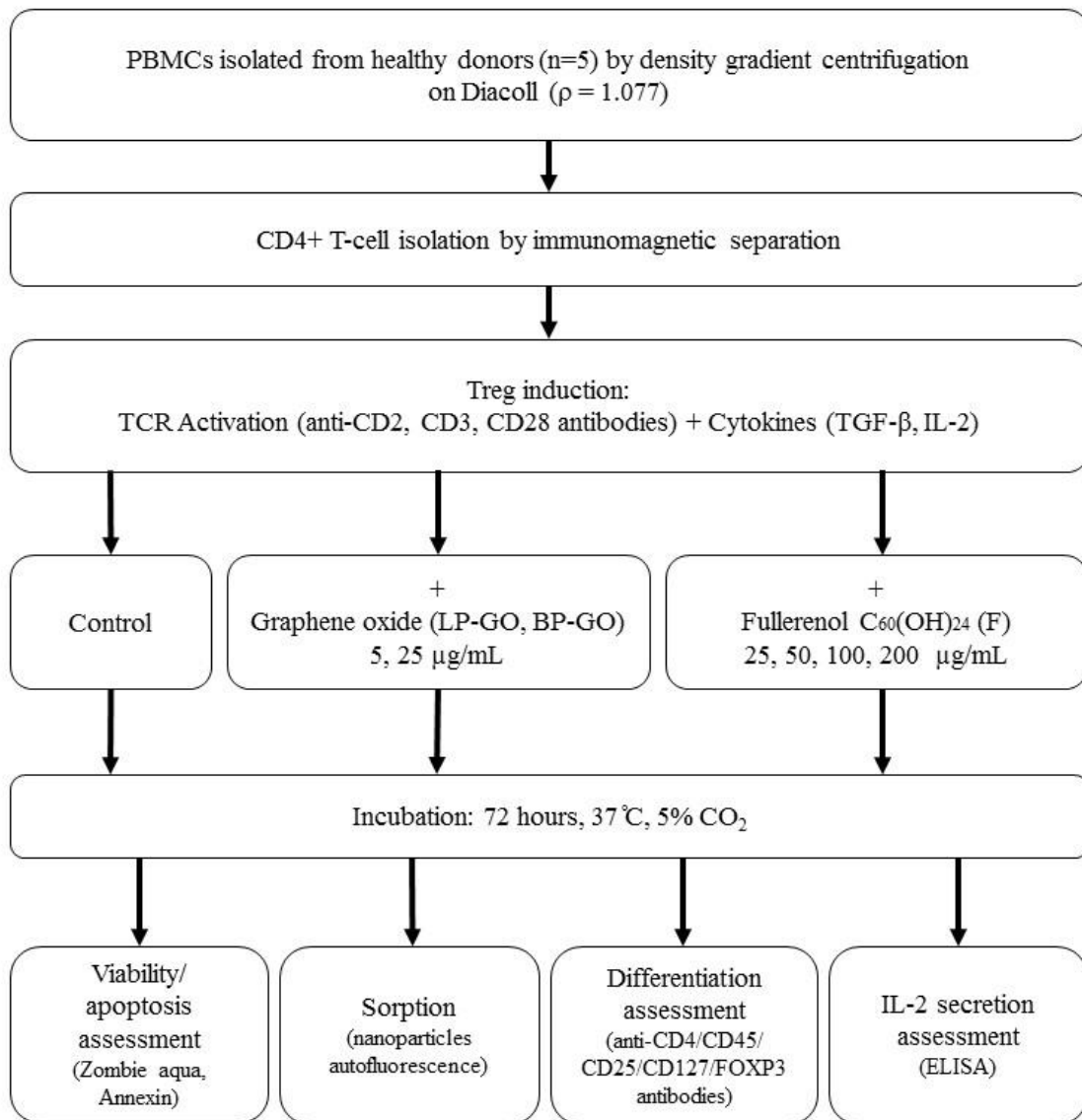

Supplement: Supplementary file 1 [file nanomaterials-16-00667-s001.zip › Supplementary S1.pdf]
